# Proprioception impacts body perception in healthy aging – Insights from a psychophysical and computational approach

**DOI:** 10.1016/j.isci.2025.113481

**Published:** 2025-09-03

**Authors:** Gaia Risso, Marion Bieri, Tommaso Bertoni, Isabella Martinelli, Giulio Mastria, Loredana Catinari, Lara Allet, Andrea Serino, Michela Bassolino

**Affiliations:** 1 Institute of Health, School of Health Sciences, HES-SO Valais-Wallis, 1950 Sion, Switzerland; 2 The Sense Innovation & Research Center, 1950 Sion and 1007 Lausanne, Switzerland; 3 MySpace Lab, Department of Clinical Neurosciences, Lausanne University Hospital (CHUV) and University of Lausanne (UNIL), CH-1011 Lausanne, Switzerland; 4 Department of Brain and Behavioral Sciences, University of Pavia, 27100 Pavia, Italy; 5 Centre for Studies and Research in Cognitive Neuroscience, University of Bologna, 47521 Cesena, Italy; 6 Department of Psychology, University of Bologna, 40127 Bologna, Italy; 7 Department of Medicine, University of Geneva, Geneva, Switzerland; 8 NeuroRehab Research Center, Service Universitaire de Neuroréhabilitation (SUN), Lausanne University Hospital (CHUV) and University of Lausanne (UNIL), Lausanne, Switzerland

**Keywords:** Neuroscience, Cognitive neuroscience, Techniques in neuroscience

## Abstract

The way we perceive our body and its dimensions depends on how our brain combines multisensory information. As the human sensory system declines with age, we hypothesize that body perception may change during aging. We investigated this hypothesis by comparing body ownership (BO) and upper limb perceived dimensions (mBR) in young and older individuals (>65 years). We used computational and psychophysical methods to quantify alterations in mBR and BO, and modeled their relationship with sensorimotor and cognitive factors. Results revealed altered body perception in healthy older adults, with significant underestimation in arm dimensions and increased feeling of ownership over an incongruent virtual hand, incorporating it into their motor plans. Reduced abilities in localizing one’s own body in space (i.e., proprioception) emerged as a common factor influencing both BO and mBR. These findings pave the way for stimulation strategies to maintain or restore body perception in aging.

## Highlights

• Aging increases ownership for incongruent body signals affecting motor plans
• A Bayesian model accounts for altered body ownership via proprioceptive decrease
• Age-related limb underestimation is linked to proprioceptive and cognitive decline
• Proprioception decline alters multiple dimensions of body perception in aging

## Graphical abstract

**Figure:**
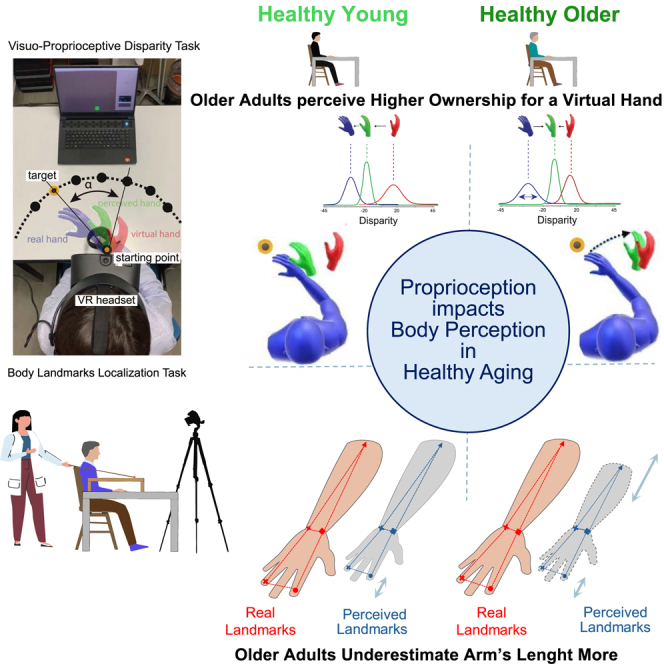

## Introduction

The human sensory systems decline throughout the lifespan, encompassing multiple sensory modalities,^1^ e.g., vision,^2^ hearing,^3^ taste, smell,^4^ tactile and proprioceptive sensitivity.^5^^,^^6^ Aging also impacts how these different modalities are integrated (see^7^^,^^8^ for a review). Coherent multisensory integration of bodily cues during daily life interactions is considered a necessary building block for different aspects of bodily experience. This includes the natural sense of body ownership (BO),^9^^,^^10^^,^^11^ i.e., the experience of the body as our own, as well as the metric body representation (mBR),^10^ which is continuously updated to encode the size and shape of various body parts—an essential process for accurate motor control.^12^^,^^13^

In pathological conditions, abnormal perceptions of the BO and mBR can be directly caused by a lack or altered flow of sensory, in particular proprioceptive information, and/or motor commands to/from the brain due to central or peripheral lesions (e.g., stroke or deafferentation),^14^^,^^15^^,^^16^^,^^17^^,^^18^ or structural damage (e.g., amputees).^19^^,^^20^^,^^21^^,^^22^ Considering the well-documented deterioration of sensorimotor processes, including proprioception^5^^,^^6^ during physiological aging,^7^^,^^8^ an emerging view suggests that possible changes in BO and mBR can also occur in healthy older adults.^7^^,^^23^^,^^24^^,^^25^

However, evidence in this direction is scarce. Previous studies investigating age-related changes in BO mainly used the RHI.^23^^,^^24^^,^^26^^,^^27^ In this paradigm, the illusion that a rubber hand belongs to the participants’ body is induced by manipulating the congruency between the visuo-tactile stimulation of the participants’ real limb (hidden from sight) and a rubber hand.^28^ Despite recent psychophysics and Bayesian modeling studies on healthy adults demonstrated that less reliable proprioception can lead to stronger ownership over fake hands, highlighting the impact of somatosensory information flow on body perception,^29^^,^^30^ the results on aging are heterogeneous. Judgments on rubber hand ownership have been reported to show no significant differences between seniors and young adults in some studies.^26^^,^^27^ However, other studies have found significantly decreased (i.e., lower illusion)^23^^,^^24^ ownership in seniors, while some report significantly increased (i.e., higher illusion) ownership during aging.^23^^,^^24^ The heterogeneity in the results may be related to the intrinsic limitations in the experimental protocol, mainly based on subjective questionnaires and the absence of specific evaluation of unimodal and multisensory underlying factors. Thus, whether and how BO is affected during aging is still a matter of debate, and the origin of these age-related changes, if any, has not been systematically investigated.

Similarly, only two studies investigated mBR during aging. These works were consistent in demonstrating a shortening in the perceived arm length^25^^,^^31^ in older adults. However, also in this case, the underlying factors contributing to these changes have not been explored due to the lack of evaluations of related sensorimotor or cognitive functions.

Furthermore, studies on body representations in the physiological aging often lack rigorous inclusion criteria in sample selection, which are necessary to rule out clinical conditions commonly found in older adults that could potentially influence the results, e.g., dementia or sensorimotor frailty. Thus, the literature offers still limited and inconclusive evidence on the alterations of body representation and the potential associated factors in healthy older adults. Bodily awareness is a central component of the human mind, with implications for sensory, motor, and cognitive systems. Without accurate knowledge about body perception during aging and its related factors, we lack of potential strategies to counteract its alterations, with an impact on the quality of life and autonomy.^7^ Accordingly, this study aims to (i) quantify age-related alterations in BO and mBR, as well as (ii) the sensorimotor and cognitive factors underlying those distortions.

To these aims, we performed an accurate screening of the older adults’ sample to ensure that only seniors in a state of cognitive and sensorimotor wellbeing were enrolled.

We adopted a recently proposed approach to overcome the limitations of the RHI-paradigm^23^^,^^24^^,^^26^^,^^27^ previously employed in aging. Unlike the classic RHI,^11^^,^^28^ our task used visuo-proprioceptive instead of visuo-tactile information, and it is based on an active, multisensory task. This new approach exploits a Bayesian causal inference framework, in which the likelihood of a limb belonging to one’s body is estimated based on the integration of multisensory body information, depending on the precision of visual and proprioceptive signals. This approach allows the quantification of age-related alterations in BO and the modeling of the underlying uni-/multi-sensory and cognitive components.^29^^,^^30^^,^^32^^,^^33^^,^^34^^,^^35^

To quantitatively assess mBR, we applied the Body Landmarks Localization task^25^ and a comprehensive battery of tasks assessing sensorimotor and cognitive functions to capture underlying factors that might explain any detected alterations. We focus on those abilities that have been previously suggested to likely contribute to mBR, i.e., tactile acuity,^36^ proprioception^37^ and cognitive functions,^38^^,^^39^ and are likely subjected to decline in aging.

In line with previous studies documenting alterations in body representations in individuals with proprioceptive impairments,^18^^,^^19^^,^^40^^,^^41^ and considering the physiological decline of proprioception during aging,^5^^,^^6^ we expect i) to identify altered BO and higher mBR distortions in older adults than in young participants and ii) to observe a role of proprioception in explaining these alterations.

## Results

Of the initially contacted older participants (N = 31), 25.81% were excluded based on the screening with ad hoc batteries for cognitive deterioration (MOCA^42^), sensorimotor frailty (according to Fried criteria^43^), and depression (Geriatric Depression Scale^44^). The final sample comprised 23 healthy older individuals (HO; 8 males; mean age: 73.82 ± 6.36). For details on the inclusion criteria for older adults, refer to Table 1 (additional info also in the Supplementary Material, including Table S1). In addition, 23 healthy young adults (HY; 12 males; mean age: 27.17 ± 4.27) participated in the experiment as a control group.

**Table 1:** Comprehensive screening assessment for older adults

Health Indicator	Mean(±SD)	Range (min-max)	CUT-OFF
GDS	3.30 (±2.99)	0–30	>20
MOCA	French speakers: 27.88(±1.17)	0–30	French speakers: <26
	Italian Speakers: 3.83(±0.41)	0–4	Italian speakers: <2
Fried Frialty Index	0.57(±0.66)	0–5	>3
Flanders	9.9(±0.30)	0–10	/

The table presents the mean values of various health indicators used for the screening of older participants enrolled in the study. We included the Geriatric Depression Scale (GDS), the Montreal Cognitive Assessment (MOCA, following the national validations: equivalent scores for the Italian speakers; total corrected score for the French speakers), and the Fried Frailty Index. None of the participants included reported results below the corresponding cut-off in any of these scales. The Flanders score evaluates handedness, ranging from 0 to 10. A score of 10 indicates strong right-handedness, 0 indicates strong left-handedness.^43^ Further details and references are reported in the STAR Methods section.

### Older adults show a higher tendency to experience ownership towards a virtual hand

**Figure 1:**
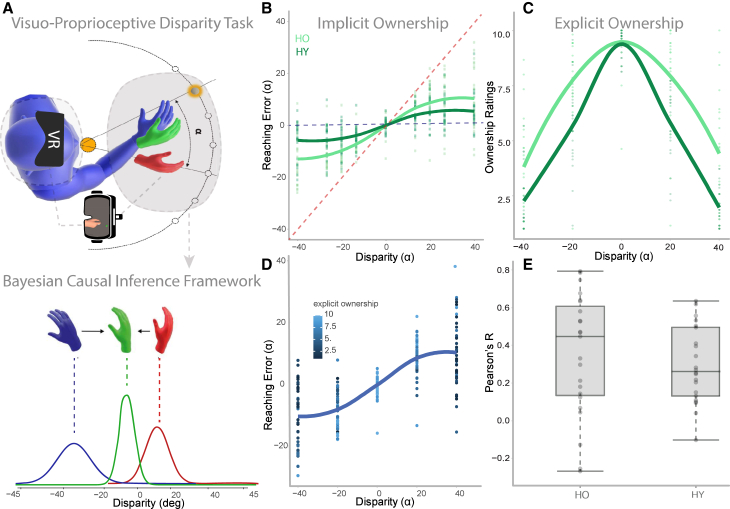
Visual proprioceptive disparity setup and results (A) Experimental setup: during the visual proprioceptive disparity task (VPD) older (*N* = 23) and younger (*N* = 23) participants are required to perform a reaching movement from the starting position to one of the seven possible target positions. The participants observe a virtual hand (in red in the figure) which in different trials, could either be in a position congruent with the real hand (0 disparity; in blue in the figure and hidden from the view in reality) or in an incongruent position at a certain angle α of disparity (7 possible angles from α = ± 40°; where positive disparities correspond to a clockwise rotation of the virtual hand with respect to the real one, while counterclockwise rotation is provided with negative disparities). On each trial, the difference between the real hand (in blue) and the movement made by the participant (in green) is calculated (reaching error). No feedback about the errors is provided to avoid corrections. VPD data were fitted with the Bayesian causal inference model (Bayesian CI). Panel A bottom row shows the hand position estimates according to the Bayesian CI model as a function of spatial disparities between the visual-virtual (red) and proprioceptive-real (blue) hands. According to the Bayesian CI framework, the final reached position (in green) is a weighted average between visual and proprioceptive guidance. The relative weight of vision and proprioception will depend on their relative precision and on the *a priori* probability computed by the brain that the virtual hand is considered as one’s own. Figure adapted from.^32^ (B) Older adults (HO) show higher implicit ownership toward the virtual hand than younger adults (HY). The plot shows the mean values of the reaching error of each participant for each level of disparity between the real and virtual hand. The horizontal blue dotted line at zero error represents the participants’ performance if they consider only the proprioceptive signal (i.e., if they reach the target following their true hand, in blue). The red diagonal dotted line represents the participants’ performance if they follow only the visual signal (i.e., if they reach the target following the virtual hand in red). Older adults' (in light green) performance is closer to the red diagonal line thus suggesting that they weigh the visual signal more and tend to follow the virtual hand more than young adults particularly for negative disparities (linear mixed model (LMM): β = −0.16, 95% CI [−0.18, −0.15], t(6034) = −19.70, *p* < 0.001; Std. beta = −0.36, 95% CI [−0.40, −0.33]). (C) Older adults show higher explicit ownership toward the virtual hand than younger adults. The plot shows the mean ownership ratings reported for the virtual hand by each participant for each level of disparity. As expected, in both groups, higher levels of ownership toward the virtual hand are reported for lower visuo-proprioceptive disparities. However, older adults (dark green) reported a higher level of ownership toward the virtual hand for each level of disparity (LMM: β = −1.63 | 95% CI [−1.97, −1.29], group: t(1601) = −9.48, *p* < 0.001; Std. beta = −0.47, 95% CI [−0.57, −0.37). (D) Relationship between explicit judgments and implicit behavior. The plot shows the mean values of the reaching error of each participant (HO and HY) in the third and final block, during which participants also reported ownership ratings for each trial. As expected, higher ownership ratings (represented by light blue dots toward 10, in the legend) were reported at lower disparities. Noteworthy, higher ownership ratings are also reported for higher disparities by participants showing higher reaching errors (i.e., following more the virtual hand), showing the relationship between implicit and explicit ownership experience. (E) Explicit and implicit ownership correlations in young and older adults. The plot shows individual Pearson’s correlation coefficients between residual reaching errors and ownership ratings calculated trial-by-trial in the two groups.

To quantitatively measure age-related alterations in BO, we applied the visuo-proprioceptive disparity (VPD) task (see Figure 1A).^30^^,^^32^ In the VPD task, participants were asked to reach a visual target while seeing a virtual hand displaced with respect to the actual position of their real, not visible, hand, with 7 possible displacement angles varying on a trial-by-trial basis. Participants were asked to reach the visual target with their real hand (i.e., following proprioceptive information). In the last of the three blocks (see STAR Methods), they were also asked to report their agreement with the statement “I felt as if the virtual hand was my hand” on a Likert scale from 1 to 10 (maximum agreement) on each trial, for the different disparity levels (see STAR Methods and Figure 1).

As visible from Figure 1C, Healthy Older adults (HO) reported significantly higher explicit ownership ratings than the younger ones (HY; Linear Mixed Model (LMM): β = −1.63 | 95% CI [−1.97, −1.29], group: t(1601) = −9.48, *p* < 0.001; Std. beta = −0.47, 95% CI [−0.57, −0.37]). As a behavioral proxy of BO,^30^^,^^32^ we analyzed the reaching error, defined as the distance between the target position and the real hand position at the end of the reaching (see STAR Methods for the details). Crucially, the reaching error covaried with the explicit ownership ratings at a trial-by-trial level (see Figure 1E), confirming that the reaching error observed in the behavioral task is effective in capturing implicitly the experience of ownership perceived by the participants (see Figures 1D and 1E).

Reaching error was higher toward the virtual hand in older adults than younger adults, and the difference was particularly significant for negative disparities (LMM: β = −0.16, 95% CI [−0.18, −0.15], t(6034) = −19.70, *p* < 0.001; Std. beta = −0.36, 95% CI [−0.40, −0.33]). As evident from Figure 1B, the reaching movements of older adults are more shifted toward the virtual hand than the movements performed by the younger ones.

Finally, in line with previous results in patients with stroke,^32^ we also analyzed explicit and implicit ownership at 0 disparity (i.e., when the virtual and the real are spatially congruent) to explore the potential disembodiment of the participants’ real hand. No evidence of disownership between HO and HY were found in either explicit ownership ratings (Wilcoxon signed-rank: z = 2.41, *p* = 0.013, HO > HY) or reaching error (z = 0.14, *p* = 0.896) at 0 disparity. Moreover, at 0 disparity, we did not observe any significant correlation between σ_PJ_ and either explicit ownership ratings [r(44) = −0.02, *p* = 0.89] or reaching error [r(44) = 0.10, *p* = 0.51]).

### Older adults show more bias in metric body representation

**Figure 2:**
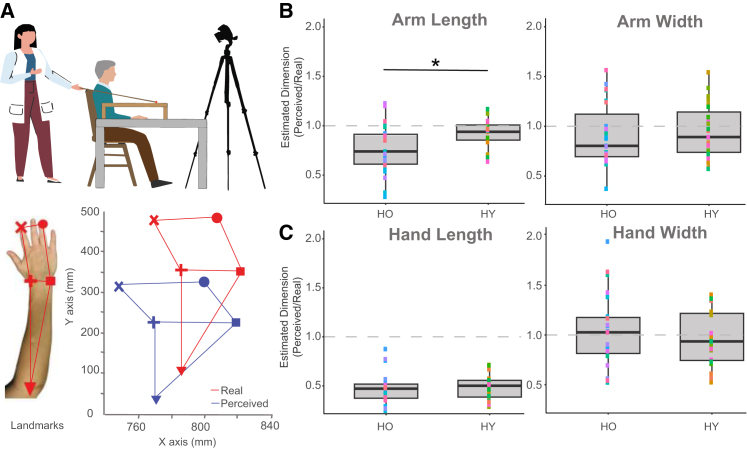
Body landmarks localization task setup and results (A) Body landmarks localization task. The top panel shows the experimental environment in which the participant is seated with the arm resting in a fixed position, hidden from the view by a table. At each trial, the participant guides the experimenter holding a stick with a marker on the perceived location. The localization is recorded thanks to a tracking system. The five landmarks that the participants were required to localize (tip of the index, tip of the ring, internal and external wrist, and elbow) are shown in the bottom row, together with a possible example of the offline reconstruction of the tracked localization of the real landmarks (in red) and the perceived landmarks (in blue). Adapted from.^18^ (B) Older adults underestimate arm’s length with respect to younger adults. The panel represents the estimated width and length (i.e., the ratio between the perceived and the real size) of the hand. Data are represented as boxplots showing the median (horizontal line), interquartile range (box), and individual data points color-coded by participant ID. Values below 1 (dashed line) indicate an underestimation, while values above 1 indicate an overestimation. While no differences can be observed for the arm width perception, older adults significantly underestimate the arm length with respect to younger ones (∗Wilcoxon signed-rank: z = −2.30, *p* < 0.05). (C) Older and younger adults show no differences in hand estimated dimensions. Data are represented as boxplots as in panel (B). No differences in the hand estimated perception (neither for length nor width) can be observed between the two groups.

To investigate alterations in mBR concerning the upper limb, we applied the Body Landmarks Localization task (BL; see Figure 2A), previously used in several studies to investigate the perceived dimensions of participants' body parts.^13^^,^^25^^,^^45^ The same 23 healthy older individuals involved in the VPD experiment and another 23 healthy young adults (HY; 8 males, mean age: 24.82 ± 4.09) participated in the experiment. Participants were asked to localize specific landmarks on the upper limb while their arm was hidden from their view, including the index finger, ring finger, internal wrist, external wrist, and elbow (Figure 2A), as previously described elsewhere.^18^^,^^25^^,^^46^ The perceived lengths of the different body parts were indirectly computed as the distance between the different landmarks, and the main output for the analyses consists of an index of estimated dimension^13^: $Estimated\;Dimension\;\left(ED\right)=\frac{Perceived\;Dimension}{Real\;Dimension}$. An index of 1 reflects a perfect perception of body dimensions (i.e., no difference between the perceived and real dimension), while <1 and >1, respectively, indicate underestimation and overestimation of perception with respect to the real body dimension. Older adults significantly underestimated their arm length (distance between wrist and elbow landmarks) as compared to younger adults (Wilcoxon signed-rank: z = −2.30, *p* = 0.021; see Figure 2B left panel). No other significant differences emerged see Figures 2C and 2B, right panel). As a general measure of distortion in mBR, we calculated an index of bias by showing how much the perceived arm dimensions differ from the actual arm dimensions independently of the direction of error (underestimation or overestimation). We computed this Estimated Error Index as follows: Estimated Error (EE) = |ED-1|. The lowest possible value is 0, indicating no bias in arm length perception, while higher EE values represent higher errors regardless of the direction. Older adults exhibit greater general distortion compared to younger individuals (z = −2.82, *p* = 0.005).

### Factors underlying age-related distortions in body perception

To better understand the sensorimotor and cognitive components at the origin of the BO alterations in older adults assessed with the VPD task, we applied the Bayesian causal inference framework.^30^^,^^33^ According to this model, sensory integration depends on: i) “bottom-up” *sensory* components, based on the weighted estimation of sensory cues (vision of the virtual hand and proprioceptive signals from one’s own hand), with relative weights proportional to their reliability; ii) top-down *priors* which represent the *a priori* probability that sensory (i.e., visual and proprioceptive) information come from the same object (that is, one’s own hand).

A higher prior will lead to higher ownership values, and thus to a higher weight of visual cues in guiding reaching movements (see Figure 1A). We modeled the task as a Bayesian causal inference process extracting sensory and cognitive components,^30^^,^^33^ as follows: i) bottom-up components as the weight given to each cue (visual: σ_v_ and proprioceptive: σ_p_); ii) top-down component computed as the prior probability of a common cause P_comPrior_ among the signals.

**Figure 3:**
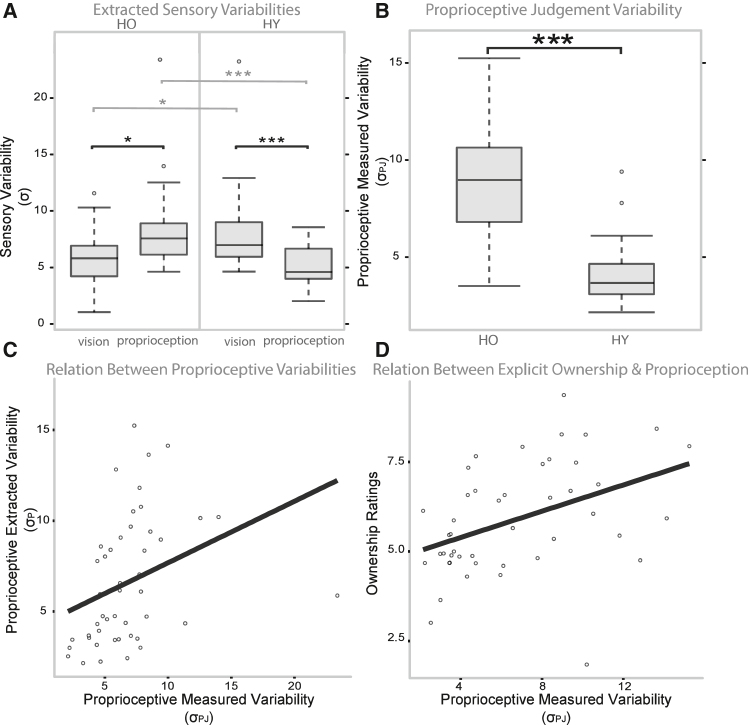
Factors underlying age-related distortions in body perception (A) Visual dominance at reaching VPD task for the older adults but not for the younger ones: The visual (σ_V_) and proprioceptive (σ_P_) variabilities of the participants extracted by the model from the multisensory reaching performance to the VPD task are shown for older adults (HO) and younger ones (HY). Higher σ values represent higher variabilities. Data are represented as boxplots showing the median (black horizontal line), interquartile range (box), and whiskers extending to 1.5×IQR. The asterisks indicate significant differences between groups (in light gray) and within groups (in black): Wilcoxon signed-rank test, ∗∗∗*p* < 0.001; ∗∗*p* < 0.01; ∗*p* < 0.05. (B) Younger adults show higher proprioceptive accuracy than older adults: The figure shows the comparison between the proprioceptive variability σ_P_ of the two groups directly measured through a purposely designed task (PJ), Wilcoxon signed-ranks test, z = −5.416, ∗∗∗*p* < 0.001. Data are represented as boxplots as in panel (A). (C) Linear relationship between the proprioceptive performance during the multisensory reaching task (σ_P_ extracted from the VPD task) and the variability directly assessed with the unisensory proprioceptive task (σ_PJ_ assessed with the PJ task; r_s_(44) = 0.554, ∗∗∗*p* < 0.001). (D) Linear relationship between the explicit judgment of ownership (mean ownership ratings) and the proprioceptive performance (σ_PJ_, variability at the PJ task; r_s_(44) = 0.477, ∗∗∗*p* < 0.001).

Related to the bottom-up component, and as visible from Figure 3A, the proprioceptive (σ_p_) [Wilcoxon Signed-Ranks Test: z = −3.797, *p* < 0.001] and visual (σ_v_) [z = −2.257, *p* < 0.05] variability of the older adults at the VPD are both significantly higher than that of the younger adults, indicating that older adults have significantly less sensory precision in their real hand localization. However, σ_p_ is significantly higher than σ_v_, meaning that for older adults’ vision is the most reliable sensory cue during the VPD task [z = −1.973, *p* < 0.05]. Conversely, in younger participants, the difference between σ_p_ and σ_v_ shows the opposite direction, with proprioception being the most reliable sensory cue [z = −3.221, *p* = 0.001]. To confirm that the older adults’ results related to proprioceptive precision extracted from the model truly reflect their proprioceptive accuracy, we used the independent, purely unisensory, tasks^30^^,^^32^ i.e., the static proprioceptive judgment task (PJ; see also the Table in the “sensorimotor and cognitive tasks’ battery” section of the STAR Methods), the Open Loop (OL) reaching task and the visuo-proprioceptive alignment Midline Judgment task (MJ). While no significant differences between groups emerged on the OL [Wilcoxon Signed-Ranks Test: z = −0.741, *p* = 0.459] and MJ (z = −0.610, *p* = 0.542]), older adults show significantly lower proprioceptive precision with respect to the younger participants at the unisensory PJ task, [z = −5.416, *p* < 0.001; see Figure 3B]. Moreover, the proprioceptive precision observed at the PJ (σ_PJ_) and the one extracted from the model (σ_p_) correlate [r_s_(44) = 0.554, *p* < 0.001]. This result shows that the multisensory behavior (from which σ_p_ was extracted) truly reflects the sensory variability observed during a unisensory proprioceptive task (σ_PJ_). Finally, to verify possible age differences related to the top-down component, we found that the P_comPrior_ is not significantly different between the two groups [z = −0.847, *p* = 0.397]. However, the proprioceptive variability σ_PJ_ of the participants significantly correlates with their explicit ownership ratings [r_s_(44) = 0.477, *p* < 0.001].

Taken together, these findings suggest that decreased proprioceptive precision, and not changes in the priors, leads to an alteration of BO during aging, as shown by an increased tendency to embody a virtual hand in older compared to younger adults.

To investigate the underlying components of age-related bias in mBR, we first aimed to evaluate the factors that have been previously hypothesized to contribute to this bias and were particularly relevant for aging.^43^^,^^47^^,^^48^^,^^49^ mBR is built up and updated through the continuous flow of sensory information about our own body from various sensory^36^^,^^45^ and motor^12^^,^^13^ sources, as well as being influenced by higher-level cognitive factors.^39^^,^^50^ For the sensory component, we evaluated tactile abilities previously found to be correlated with mBR,^36^ specifically using the two-points discrimination task. Given the importance of proprioception for the perceived dimension of the limb,^37^ but the lack of consensus on quantitative measures of proprioceptive abilities in aging,^51^ we utilized the rigorous and quantitative proprioceptive tasks embedded in the VPD protocol. These tasks targeted different components of proprioception, including static proprioception (via the proprioceptive judgment task), dynamic proprioception (evaluated through an open loop reaching task), and visuo-proprioceptive alignment (measured using a midline judgments task; see STAR Methods with table for more details). Moreover, considering the decline of cognitive flexibility,^52^ short and working memory^48^ during aging and their potential role in mBR, we included tasks to assess these functions (the trial making test, the digit span forward and backward, respectively; see STAR Methods with table for further details).^43^ After identifying these variables, we applied a Lasso (Least Absolute Shrinkage and Selection Operator) regression on the estimated arm error to select the most relevant explanatory variables, reducing the high number of potential predictors. This method is particularly effective in scenarios with many possible predictors, as it helps to ensure robust model performance even in high-dimensional datasets.^53^ Once we had selected the relevant variables using Lasso regression, we then examined the relationship between these selected factors and mBR using linear regression analysis.

The linear regression model predicting the arm length estimated error is presented in Table 2. It significantly fitted the data explaining the 53% of the variability of the model (46% R-squared adjusted; F(3,19) = 7.24, *p* = 0.002, $f^{2}$ = 1.143, see STAR Methods for more details on the variable selection) and included: i) cognitive flexibility (β = 0.10, t(19) = 3.23, *p* = 0.004), assessed with the TMT, ii) visuo-proprioceptive ability (β = 0.09, t(19) = 3.00, *p* = 0.007), assessed through the response variability of the MJ task and iii) proprioceptive ability in dynamic condition (β = 0.02, t(19) = 2.01, *p* = 0.059), assessed through the response variability of the OL task. These parameters significantly predicted the general bias in arm length, suggesting that the observed metric bias in older adults may be associated with declines in cognitive flexibility, visuo-proprioceptive alignment abilities, and dynamic proprioceptive abilities.

**Table 2:** Lasso and linear regression output

Variable	Task	Lasso Included	T-value	Regression *p*-value
Arm Tactile Acuity	Two Points Discrimination task	No	/	/
Hand Tactile Acuity	Two Points Discrimination task	No	/	/
Proprioceptive variability in static condition	Proprioceptive Judgment task	No	/	/
Proprioceptive variability in dynamic condition	Open Loop task	Yes	2.007	0.059
Visuo-proprioceptive ability	Midline judgment task	Yes	2.996	0.007
Short-term memory	Digit Span Forward	No	/	/
Cognitive Flexibility	Trail Making Test B-A	Yes	3.226	0.004

The table shows the output of the Lasso and linear regressions with the Estimated Error on the arm length as the dependent variable. The first column indicates the sensorimotor and cognitive potential predictors of bias in mBR considered in the analyses. The second column summarizes the task used to evaluate each factor. The third column indicates whether the variables resulted as explanatory variables according to the Lasso regression method to predict the Estimated Error (EE) on the arm. The fourth and fifth columns report, respectively, the t-values and the associated *p*-values of the resulting linear regression model.

## Discussion

The present results highlight significant differences between young and older adults in the experience of their own body, focusing on the two components of Body Ownership (BO) and perceived dimensions (metric Body Representation; mBR).

Our findings reveal a consistent increase in both explicit (i.e., direct ownership judgments) and implicit (i.e., the behavioral reaching error) measures of ownership toward a virtual hand in older compared to younger individuals. This indicates an increased tendency to incorporate external objects and difficulty in distinguishing them from one’s own body during aging (see^30^^,^^32^^,^^33^). Specifically, these results demonstrate that older adults show a wider spatial window in integrating visual information about the virtual hand with proprioceptive information about their real hand at higher disparities. In contrast to the classic RHI, the current approach offers the significant advantage of enabling the simultaneous testing and modeling of the underlying distinct contributions of sensory and cognitive components of BO. We hypothesize that the present findings may generalize to other protocols based on visuo-tactile stimulation, consistent with recent results obtained using a Bayesian causal inference model,^29^^,^^34^ which show that the probability of experiencing the illusion in a visuo-tactile RHI protocol increases with proprioceptive uncertainty. This is also in line with previous studies suggesting that illusory ownership similarly occurs under various combinations of multisensory information (see, for instance^54^; for a TMS version of the RHI^55^; for a robotic version of the VPD^56^). However, we cannot rule out potential differences in the magnitude of effects (i.e., explicit and implicit ownership) in the case of other sensory modalities or further changes in the experimental protocol.

The question of whether any bias in attributing ownership to a fake hand in aging is due to (i) top-down priors (e.g., perceptual priors,^11^^,^^37^ priors from learning, experience, or demand characteristics^7^^,^^24^^,^^26^^,^^57^), or (ii) changes in bottom-up sensory abilities that may occur with age,^8^^,^^27^^,^^58^^,^^59^ is still debated.^7^ Our results clearly support the second hypothesis, showing a significantly diminished ability to rely on proprioceptive cues during aging, while top-down priors do not differ between older and young participants. Differently stated, the stronger tendency of older adults to attribute ownership to a virtual hand, even when it is visually displaced from their own hand, can be attributed to their reduced proprioceptive sensitivity. This conclusion is further confirmed by the correlation between participants’ proprioceptive accuracy, assessed through an independent unisensory task, and their explicit ownership ratings during the multisensory task.

These findings in older adults align closely with recent results in post-stroke patients with proprioceptive impairments. By applying the same experimental and computational approach, the results demonstrated that patients exhibited a higher implicit (reaching error) and explicit (ownership ratings) attribution of a virtual hand to their body, specifically for the limb contralateral to the affected hemisphere. Notably, the alterations observed on the affected side in patients with stroke were more pronounced than those found here in healthy aging, and included also the disembodiment of patients’ real hand.^32^ Specifically, patients not only reported higher implicit and explicit ownership toward the virtual hand when there was a disparity with their real hand, but also, they reported less ownership toward the virtual hand when it was congruent with the real hand, i.e., at zero disparity. The latter effect was not present in the healthy participants examined in this study. Interestingly, these pronounced effects in patients with stroke were not solely explained by proprioceptive deficits but also by patients' lesions in the frontoparietal network. This suggests that BO alterations in brain-damaged patients result from two parallel mechanisms: reduced proprioceptive precision and an impaired multisensory integration due to frontoparietal network damage. Put together, the evidence collected in healthy older adults and patients with stroke suggests a crucial role of proprioception in the modulation of BO, while disownership of one’s own hand and multisensory integration deficits remain a peculiar marker of neurological conditions, likely involving lesions of the frontoparietal network. Furthermore, our findings align with the conclusions of previous studies showing that, despite the preserved multisensory integration, older individuals exhibit a greater reliance on visual inputs for hand movement perception compared to younger individuals. In agreement with our conclusions, these studies suggest that the observed shift toward a visual reliance can be explained by a decline in proprioception with age.^60^^,^^61^

We also identified alterations in the perception of body dimensions in older adults (mBR), who perceived their arms as significantly shorter than younger controls. Importantly, these findings are consistent with the two reports related to mBR during aging.^25^^,^^31^ This key finding demonstrates the robustness of our results across different samples and methods, highlighting the need to consider age-related changes in body perception.

Recent studies have investigated age-related changes in proprioceptive signals and multisensory representation of the arm, demonstrating that proprioceptive uncertainty can alter arm position perception^60^ and that older adults exhibit greater proprioceptive decline compared to tactile perception, impacting body representation and hand movement perception.^61^

However, while changes in perceived body dimensions are commonly observed also in healthy young adults,^13^^,^^25^^,^^62^^,^^63^ the driving factors of these distortions in mBR have not yet been revealed, neither in the young nor in older healthy individuals. The identification of the mechanisms underlying such a bias is an outstanding question in the recent literature on body representation, with important implications also for motor control.^37^^,^^64^^,^^65^ Our data demonstrates that during physiological aging, not mutually exclusive sensorimotor or cognitive factors contribute to the distortions in arm perception. Specifically, we found that a higher error in perceiving arm length bias during aging is predicted by worse cognitive flexibility, along with reduced skills in visuo-spatial alignment and dynamic proprioception.

Crucially, proprioceptive components emerge again as significant factors for body perception, reinforcing the importance of proprioception not only for BO but also for mBR. Specifically, our model identifies two proprioceptive factors of mBR. On the side, higher variability in a purely reaching task (in the absence of any visual feedback) is predicting a less accurate mBR in older adults. This result aligns well with previous literature suggesting a link between mBR and sensorimotor control.^12^^,^^66^ Plasticity in mBR may be related to sensorimotor experiences,^12^ such as after tool-use,^63^^,^^67^^,^^68^ immobilization,^69^^,^^70^ or in individuals with specific hand skills.^50^^,^^71^^,^^72^ Pathological mBR biases have also been linked to declines in peripheral mechanoreceptors in muscles and joints affecting proprioception,^7^^,^^8^^,^^73^^,^^74^ or to reduced sensorimotor information flow.^19^^,^^21^^,^^40^ For instance, in patients with stroke with sensorimotor impairments, the affected arm is perceived as shorter, possibly due to asymmetrical arm use and reduced sensorimotor flow.^18^ Recently, a link between distortions in mBR in young individuals and variability in proprioceptive skills has been demonstrated.^37^ On the other hand, increased variability in localizing one’s own body midline also predicts a decline in mBR accuracy with aging. Our midline judgment task required participants to indicate when a visual reference in a VR environment aligned with their midline. Participants could not see their own bodies during the task. Accordingly, this requires remapping proprioceptive information about the space and position of the body into a visual frame of reference. Being able to compute the location of an object in different reference frames is not just a theoretical exercise; it is crucial for the successful completion of different types of behavior in everyday life.^75^ For instance, when sitting at a table and looking at a cup on it, the location of the cup can be described in different reference frames. Relative to the observer’s eyes, the cup might be straight ahead, but relative to their arm on the side, the cup might be on the right. The cup’s location can also be described in a reference frame that depends on the external world rather than the location of your body, for example, relative to its position on the table.^75^ In the midline judgment task, participants need to transform proprioceptive information about their body’s position with visual information about the reference’s location to accurately determine alignment. This transformation is crucial for tasks that require precise spatial judgments, and previous studies have demonstrated its relevance to body perception. The illusory ownership over a rubber hand has been demonstrated to be strongest when multisensory information about the body is coded into a common frame of reference^76^^,^^77^^,^^78^ as when seen from a first-person viewpoint at an anatomically plausible position.^10^^,^^79^ Overall, our findings align with previous literature in indicating that poorer transformation ability, as assessed by the midline judgment task, is associated with greater errors in mBR during the BLT. This is analogous to findings in the RHI studies, where the illusion of ownership over a rubber hand is stronger when multisensory information is integrated into a common frame of reference, that is, in conditions where the transformation process between visual and proprioceptive information requires less cognitive effort. Thus, our results underscore the importance of accurate visuo-proprioceptive transformations for precise body representation.

Finally, our model suggests that less accurate mBRs are also predicted by lower performance in cognitive flexibility in older adults. Previous studies already suggest that distortions in mBR might be related to several general executive functions needed to combine visual memory of the location of different landmarks,^39^^,^^80^ or to update the information coming from the body.^38^^,^^81^^,^^82^ However, here we found that only cognitive flexibility measured by the TMT test correlates with the mBR bias. The poorer cognitive flexibility ability might reflect a higher level of difficulty in switching between multiple (visual and proprioceptive) online information during the tasks. Nevertheless, the significant correlations between cognitive flexibility and mBR accuracy should be interpreted with caution, as they may reflect broader changes in cognitive functions impacting on sensorimotor abilities rather than direct mechanistic links (see limitations of the study section).

### Limitations of the study

This study investigates differences between two well-separated age groups of young and older adults. The significant differences observed between these groups support the importance of extending the present findings. Future studies should include a broader range of age groups to capture the full spectrum of age-related differences and enhance the generalizability of the findings, providing a comprehensive understanding of how healthy aging impacts body perception. Furthermore, the cross-sectional design of this study provides a snapshot of body perception at two distinct ages and does not describe the dynamics of its evolution within each cohort that can be further addressed by future longitudinal studies.

Furthermore, while for the modeling of body ownership we exploited a Bayesian approach to data analysis, to predict mBR biases we used a correlational approach. Despite using specific techniques for appropriate variable selection and modeling, such an approach might have limitations due to confounding variables or a limited sample. Correlational analyses cannot indicate a direct causal mechanism, and future studies should investigate if other factors can mediate the observed relationship, particularly for higher cognitive functions. The Bayesian approach we applied to the illusion of ownership could also be transposed to the mBR with appropriate adaptations of the employed experimental task. This would allow a better understanding of the mechanisms underlying mBR bias.

It is important to underline that the differences highlighted in the current study relate to the comparison between young adults and a group of older adults in good psychophysical health. Our study excluded cognitively impaired or frail individuals. This means that the changes in body perception observed in this study reflect a condition affecting the entire population over the course of natural aging.

Integrating body perception assessments with traditional sensorimotor evaluations could provide a more comprehensive approach to timely monitoring and supporting healthy aging. This is particularly relevant considering that the sensorimotor and cognitive factors we showed to underlie body representations are subject to decline in older adults. One could hypothesize that mBR and BO might become progressively more distorted during aging, particularly in the presence of preclinical or pathological conditions affecting the underlying sensorimotor and cognitive functions. This might lay the groundwork to generate new prevention and stimulation strategies in typical aging, not only focused on sensorimotor functions, but also on body perception. In line with this, new methods for the rehabilitation of distortions in body representation have recently been proposed^20^^,^^40^ and tested in clinical populations,^18^^,^^19^^,^^21^^,^^46^ but not yet extended to support wellbeing in seniors.

In conclusion, our findings highlight the importance of proprioception in body representation in healthy older adults and suggest that addressing subtle changes in body perception may open new avenues for promoting healthy aging and preventing functional decline.

Overall, this study identified reduced abilities in older adults in various components of proprioception, which involve localizing one’s own body in space, as playing a crucial role in maintaining and updating an unambiguous perception of one's own body across different components, including mBR and ownership, in line with our initial hypothesis. With respect to other exteroceptive sensations such as vision and hearing that track mainly stimuli related to external objects, proprioception is strongly associated with one’s own body and its movements^83^^,^^84^^,^^85^^,^^86^ in typical conditions, and alterations in ownership in patients with stroke.^31^^,^^32^^,^^41^

## Resource availability

### Lead contact

Further information and requests for resources should be directed to Michela Bassolino: michela.bassolino@hevs.ch.

### Materials availability

This study did not generate new unique reagents or custom modeling scripts.

### Data and code availability

• Data: The data of this study are available from the authors upon request.
• Code: This article does not report the original code. The computational model used is described in *Bertoni* et al. *The self and the Bayesian brain: Testing probabilistic models of body ownership through a self-localization task. Cortex 167, 247–272 (2023)* and the related software are available therein.
• Additional Information: Any additional information required to reanalyze the data reported in this article is available from the lead contact upon request.

## Acknowledgments

The authors express gratitude to the younger and older healthy controls whose participation significantly contributed to advancing our research. We thank Pro Senectute Vaud and Avivo Lausanne for their support in disseminating recruitment flyers to older adults, and Brunella Donno for the body landmark illustration (panel A) in Figure 2. This work was supported by 10.13039/501100023177the Fondation Pierre Mercier pour la Science grant to MB. The founders had no role in study design, data collection and analysis, decision to publish, or preparation of the article.

## Author contributions

M.B., A.S., and L.A. designed the study, M.B. and A.S. supervised the experiments and analyses. G.R. performed the initial experiments, analyzed the data, conducted the statistical analysis, made the figures, and wrote the article with M.B. M.Bi., and I.M. recruited and tested the healthy participants. T.B. and G.M. contributed to the modeling of ownership evaluation. M.B., G.R., M.Bi., and I.M. L.C. selected the questionnaires and tests used to assess the participants’ health status and cognitive performance. All authors reviewed and approved the final article.

## Declaration of interests

The authors declare no competing interests.

## STAR★Methods

### Key resources table

**Table:** 

REAGENT or RESOURCE	SOURCE	IDENTIFIER
**Software and algorithms**		

MATLAB	Mathworks	R2024b, https://it.mathworks.com/products/matlab.html
R	R Project for Statistical Computing	R4.4.1, https://www.r-project.org
Virtual reality assessment	Bertoni, T. et al. The self and the Bayesian brain: Testing probabilistic models of body ownership through a self-localization task. *Cortex* 167, 247–272 (2023).	OSF: https://osf.io/azh8p/
Code for model fitting	Bertoni, T. et al. The self and the Bayesian brain: Testing probabilistic models of body ownership through a self-localization task. *Cortex* 167, 247–272 (2023).	OSF: https://osf.io/azh8p/
BADS optimization tool	Acerbi, L. & Ma, W. J. Practical Bayesian Optimization for Model Fitting with Bayesian Adaptive Direct Search. (2017) https://doi.org/10.48550/ARXIV.1705.04405.	https://github.com/lacerbi/bads

### Experimental model and study participant details

#### Subjects

Older adults. A group of 26 older participants were recruited in Switzerland or Italy (4 participants). All participants were of European ancestry, identified as White/Caucasian, and spoke either French or Italian as their primary language. Participants with cognitive impairments (i.e., equivalent scores <2 for the Italian speakers^87^^,^^88^; total score cutoff<26 for the French speakers^42^) assessed by the Montreal Cognitive Assessment (MOCA^42^) were excluded from the experiment. The Montreal Cognitive Assessment (MOCA) has a minimum score of 0 and a maximum score of 30, and it assesses different cognitive domains, including attention, concentration, executive functions, memory, language, visuospatial skills, and orientation. Additionally, individuals with severe depression (score >20) assessed by the Geriatric Depression Scale^44^ (min score: 0, max score: 30), or identified as fragile according to the criteria of Fried^43^ were excluded from the experiment. The Fried Frailty Phenotype is a widely used tool for assessing frailty in older adults,^43^ identifying frailty based on five criteria. An individual is considered frail if they meet three or more of these criteria. 1) Involuntary weight loss of at least 5 kg in the last 12 months. A score of 1 is assigned if more than 5 kg were lost in the last year, 0 if not. 2) Fatigue and exhaustion. This is assessed through self-evaluation on a four-point scale, based on the individual’s perception over the past week. If they felt that everything was an effort or that it was impossible to get going on most days, they were asked to rate their experience. If the response to either of these questions was “often” or “most of the time” (corresponding to a Likert scale rating of 3 or 4), 1 point was assigned. 3) Weaknesses, assessed by measuring a 20% decrease in Isometric Arm Grip Strength using a dynamometer, adjusted for gender and BMI. If the adjusted strength falls below the normative threshold 1 point is assigned; otherwise, 0 points are given. 4) Slowness based on the time taken to walk 4-metres, adjusted for gender and height. Slower times indicate frailty. If the normalized score is below the normative threshold, 1 point is assigned; otherwise, 0 points are given. 5) Decreased physical activity based on the number of kilocalories expended per week (<383 kcal for men and 270 for women). It relies on self-reported frequency and intensity of physical activities on a 6-point Likert scale. If the response is "no activity" or "mostly sedentary" (corresponding to Likert scale ratings of 1 or 2), 1 point is assigned; otherwise, 0 points are given.

The final group consisted of 23 (8 males) with a mean age of 73.82 ± 6.36. The participants were right-handed as confirmed by the Flanders Handedness Survey,^89^ where participants rate their preference for using the right or left hand in ten different situations. A score of 10 indicates strong right-handedness, while a score of 0 indicates strong left-handedness. Individuals had normal or corrected-to-normal vision, no psychiatric or neurological deficits, no pain or sensorimotor pathologies in the upper limbs, or fractures in the previous 12 months. Older participants were tested in two different experimental sessions to avoid fatigue during which all tasks were performed (VPD task; BL task; and all the sensory, cognitive, and motor tasks reported in the Table in the “sensorimotor and cognitive tasks’ battery” section of the STAR Methods).

Young controls. For practical reasons, two different groups of 23 younger adults performed respectively the VPD or the BL task. Related to the VPD task, the power analyses in^30^ indicates that a sample of 15 participants is appropriate to allow the statistical power necessary to see a correlation between the proprioceptive precisions measured through independent tasks and those fitted from the causal inference model (see below). Considering the BL task, the power analysis reported in previous studies a sample of 21 subjects is required to detect modifications in perceived dimensions of upper-limbs.^25^^,^^63^. To be conservative, we recruited 23 participants for both groups. All the participants were adults with no history of psychiatric, neurological, pain, or sensorimotor known deficits.

Specifically, for the VPD task, we recruited 23 healthy controls (12 male; mean age: 27.17 ± 4.27), while for the BL task, we recruited a second group of 23 participants (8 male, mean age: 24.82 ± 4.09). All participants provided written informed consent prior to their inclusion in the study and were tested in Switzerland or Italy according to the agreement given by the ethics committees CER-VD Canton de Vaud (project identifier: 2017-01588) and Politecnico di Milano Ethical Committee, amendment approval 11/2024.

### Method details

#### Visual proprioceptive disparity (VPD) task

The same procedure reported in previous works^30^^,^^32^ was here used for the VPD task. During the VPD task participants sat in front of a table wearing an Oculus Rift S Virtual Reality system, with their arm resting on the table while holding a motion controller (Oculus Touch) in their right hand. The real hand of the participants was hidden from their view while they observed a realistic hand in virtual reality moving coherently with the held controller. The task consists of a reaching movement from a starting position toward one of 7 possible targets (white spheres with 3 cm diameter) arranged at 0°, ±15°, ±30°, ±45° with respect to participants’ midline on an arc with radius equal to each participant's maximum reaching distance.

The starting and target positions were fixed at the beginning of the experiment and calibrated before starting to be tailored to each participant, with the starting position close to the body and approximately at the midline of the participants’ body, on the table, and the reaching position corresponding to each participant’s maximum reaching distance. The position of the head was also calibrated in such a way that the task did not proceed unless the participants were able to see the targets with the head in a centered position.

In different trials, the spatial congruency between the position of the real hand of the participants, as signaled by proprioception, and the visual feedback from the virtual hand was manipulated, by introducing 9 possible disparities, i.e.: 0° (no disparity), ±13.3°, ±20.0° ± 26.6° or ±40°. For positive disparities, the virtual hand is moved in a clockwise (CW) direction with respect to the real hand, while for negative directions the virtual hand is shifted in the counterclockwise direction (CCW) with respect to the real hand. The task consisted of three experimental blocks. The first two blocks included the disparities 0°, ±13.3°, ±26.6° or ±40° repeated for each target (7 disparities x 7 targets). To include a measure of the subjective ratings of ownership at the end of each reaching movement the last block was shortened to limit the experiment duration. Accordingly, for each target, the last block included disparities equally spaced from 0°, ±20° or ±40° (5 disparities x 5 targets). Each block lasted approximately 7 min, and the total VPD lasted about 30 min.

At the beginning of each trial, while the real hand was in the starting position, the virtual hand was rotated by one of the possible disparity angles for 1 s, and the visual gray target appeared. After 1.5 s the target was turned green, and it represented the “go” signal for the participants who were instructed to start their reaching movement. Movement of the hand outside the starting position at any time before the “go” cue would automatically restart the trial. Participants were instructed to reach the target with their real (proprioceptive) hand and return to the starting position. During the last block (the third one), at the end of each trial, participants were requested to verbally report their subjective feeling of ownership for the virtual hand, evaluating their agreement with the statement “I felt as if the virtual hand was my hand” on a 1 (minimum agreement) to 10 (maximum agreement) Likert scale.

#### Bayesian causal inference model

In the present work, we aimed to provide empirical evidence for the hypothesis that body ownership reshapes during aging. Previous works provided empirical evidence for the hypothesis that body ownership emerges from a Bayesian causal inference process.^30^^,^^33^^,^^34^ According to this framework, humans perceive their bodies through multiple senses, which often provide them with redundant information.^90^^,^^91^^,^^92^ The brain uses this redundancy as an advantage and merges the information from different sensory modalities into a robust unambiguous perception.^90^ This mechanism of integration is defined as optimal because it produces not only lower, but the lowest variance estimate that can possibly be achieved by the Nervous System. Applied to our case of visuo-proprioceptive integration, and translated to mathematical terms, Bayesian probability theory states that the best way to interpret a stimulus S_VP_ is by performing a weighted estimation between the available visual S_V_ and proprioceptive S_P_ cues, where the weights should be proportional to the reliability of the cues^90^:

(Equation 1) $$S_{VP}=w_{V}S_{V}+w_{P}S_{P}$$

where the weights, visual weight, W_V_, and proprioceptive weight, W_P,_ sum up to unity (W_V_ + W_P_ = 1) and should be proportional to the reliability R (i.e., the inverse of the noise of the corresponding cue $R_{i}=\frac{1}{\sigma_{i}^{2}}$ ) of the stimulus:

(Equation 2) $$w_{V}=\frac{R_{V}}{R_{V}+R_{P}}\text{and}\;w_{P}=\frac{R_{P}}{R_{P}+R_{V}}$$

Assuming that the unimodal cues are independent, normally distributed, and originating from the same unique object of perception (i.e., forced fusion; FF), the formula that an optimal brain is expected to apply to estimate hand position is:

(Equation 3) $$x_{FF}=x_{V}\frac{R_{V}}{R_{V}+R_{P}}+x_{P}\frac{R_{P}}{R_{P}+R_{V}}$$

where X_FF_ is the final estimate of hand position (i.e., the one used to calibrate reaching movements in our task), and x_v_ and x_p_ are the unimodal (and noisy) position estimates. However, one of the above assumptions is non-trivial. Indeed, during everyday life situations it is not always reasonable to integrate sensory signals. For the signals to be integrated the brain must know exactly which signals are derived from the same object or event. This is a form of correspondence problem, which has to be solved before the signals can be integrated to determine whether the object of perception is a part of one’s own body or not.^90^^,^^93^^,^^94^ According to Bayesian Causal Inference models, also this additional process can be described as a probabilistic event, resolved by the brain by statistically inferring the probability (P_com_) that an object is one’s own hand. Accordingly, the sensory cues involved in the process should be weighted not only according to their reliability (as in Equation 3) but also considering the probability P_com_ that the final estimate corresponds to one’s own hand. Thus, the equation describing the final estimate of hand position according to the Bayesian Causal Inference ($x_{BCI})$ can be refined as follows:

(Equation 4) $$x_{BCI}=P_{com}x_{FF}+\left(1-P_{com}\right)x_{p}$$

The probability P_com_ of one object (i.e.,: the virtual hand) to one’s own hand varies from 1 corresponding to the certainty that the virtual hand is one’s own to 0 corresponding to the certainty that the virtual hand is not one’s own. In the model, P_com_ variations would essentially depend on the spatial disparity between its location and the proprioceptively perceived hand location:

(Equation 5) $$P_{com}=f\left(\left|x_{v}-x_{p}\right|\right)$$

According to this function, it is clear that P_com_ depends on visuo-proprioceptive disparity (|x_v_-x_p_|). Second, P_com_ is maximal at zero visuo-proprioceptive disparity and decreases as disparity increases. Third, the less vision and/or proprioception are precise, the larger the disparity will be needed by the brain to detect an incongruence. Hence, if visuo and/or proprioceptive precision is reduced, P_com_ will decrease less rapidly as disparity increases, leading to embodying the virtual hand up to larger levels of discrepancy. Fourth, the larger the prior common cause is, the larger P_com_ is at all disparities, also leading to an enhanced tendency to embody and follow the virtual hand in reaching.

To obtain the equations of the Bayesian CI model, we start from the sensory component, by modeling the sensory stimuli underlying those congruencies, i.e., the joint probability distribution of physical stimuli and their associated neural representation. The relevant physical variables considered in the VPD task consist of the true positions of the real s_p_ and of the virtual s_v_ hand (expressed in degrees from the shoulder). At each trial, the virtual hand seen by the participant might be considered as “being” or “not being” the participant’s hand. In Bayesian framework it means that s_v_ and s_p_ may have one same physical cause (C = 1) or two different (C = 2). Where C is drawn from a Bernoulli distribution with probability P_π_:

(Equation 6) $$P\left(C=1\right)=P_{\pi }\left(1\right)$$

If C = 1, then s_v_ = s_p_ = s meaning that the visual and proprioceptive position of the hand is the same and is drawn from a uniform distribution on the −90/90° range, approximating the set of reachable angles. If C = 2, s_v_, s_p_ are drawn independently from the same uniform distributions.

In order to simulate variability in sensory inputs, Zero mean Gaussian noise was added to the sensory inputs true positions, to model the neural noise in sensory inputs encoding. That is, the internal representations of s_v_ and s_p_ become respectively x_v_ = s_v_ + N(0, σ_v_), x_p_ = s_p_ + N(0, σ_p_), and σ_v_ and σ_p_ therefore represent the visual and proprioceptive information uncertainty about hand position.

Given this information, the Bayes theorem allows to compute the posterior distributions for the positions of the stimuli and the number of underlying causes that an ideal observer would compute. So, starting from the number of causes of the observed stimuli, we have:

(Equation 7) $$P_{\text{com}}=P\left(C=1\vee x_{p},x_{v}\right)=\frac{P\left(x_{p},x_{v}\vee C=1\right)P\left(C=1\right)}{P\left(x_{p},x_{v}\vee C=1\right)P\left(C=1\right)+P\left(x_{p},x_{v}\vee C=2\right)P\left(C=2\right)}$$

In our experimental setup, P_com_ translates to the probability that the virtual hand is one’s own, where higher probabilities correspond to higher levels of subjective ownership. Accordingly, if the region outside the −90 to 90° range contributes negligibly to the integral, the likelihood functions defined by our generative model are:

(Equation 8) $$P\left(x_{p},x_{v}\vee C=1\right)=\frac{1}{\alpha }\exp\left[-\frac{1}{2}\frac{{\left(x_{v}-x_{p}\right)}^{2}}{\sigma_{p}^{2}+\sigma_{v}^{2}}\right]≝\;\frac{1}{\alpha }\;e^{\frac{-\delta_{s}^{2}}{2\sigma_{s}^{2}}}$$

(Equation 9) $$\alpha =\iint_{-90}^{90}P\left(x_{p},x_{v}\vee C=1\right)\approx 180\sqrt{2\pi \left(\sigma_{p}^{2}+\sigma_{v}^{2}\right)}$$

where α is the normalization constant, δ_s_ denotes the spatial visuo-proprioceptive disparity, and σ^2^_s_ is a short form for the sum of the uncertainties on visual and proprioceptive positions. When there are two separate causes, we simply have:

(Equation 10) $$P\left(x_{p},x_{v}\vee C=2\right)=\frac{1}{180^{2}}$$

Therefore, the probability of common cause is given by:

(Equation 11) $$P_{\text{com}}=P\left(C=1\vee x_{p},x_{v}\right)=\frac{P_{\pi }\frac{1}{\alpha }e^{\frac{-\delta_{s}^{2}}{2\sigma_{s}^{2}}}}{P_{\pi }\frac{1}{\alpha }e^{\frac{-\delta_{s}^{2}}{2\sigma_{s}^{2}}}+\left(1-P_{\pi }\right)\frac{1}{180^{2}}}$$

The final estimate of hand position Ŝ_p_, is obtained using the same inference process. Indeed, if P_com_ = 0, visual information should be ignored, and Ŝ_p_ should reduce to the pure proprioceptive estimate. If P_com_ = 1, visual and proprioceptive information should be integrated, and Ŝ_p_ should correspond to the “classical” forced fusion estimate^90^ in which inputs are weighted by their inverse precisions. In all the other general cases of “intermediate” P_com_, Ŝ_p_ should then be the average of the proprioceptive and forced fusion estimates, weighted by P(C = 0) and P(C = 1) respectively.

(Equation 12) $${Ŝ}_{p}=P\left(C=1\vee x_{p},x_{v}\right)\frac{\sigma_{p}^{2}x_{v}+\sigma_{v}^{2}x_{p}}{\sigma_{p}^{2}{+\sigma }_{v}^{2}}+P\left(C=2\vee x_{p},x_{v}\right)x_{p}$$

In our framework, Ŝ_p_ values should be used when computing motor commands to reach a target in the presence of visuo-proprioceptive disparity, so the reaching error will be inversely proportional to the difference between the true hand position, S_p_, and its estimate Ŝ_p_. Then, equation^31^ can be used to formulate behavioral predictions given arbitrary true positions of the stimuli (s_v_, s_p_), and individual values of sensory precisions and common cause prior (σ_v_, σ_p_, P_π_). To do so, noisy neural representations x_v_ and x_p_ are generated based on the selected values of s_v_ and s_p_, and inserted in Equation 12 to compute Ŝ_p_. This process corresponds to simulating one behavioral trial.

#### Bayesian causal inference framework transposition to visual proprioceptive disparity (VPD) task

When reaching a target in the VPD task, subjects combine spatial proprioceptive information about the position of their real hand with incongruent spatial information from the virtual hand visually perceived. According to the Bayesian causal inference framework, the final reached position toward the target will be a weighted average between pure visual and pure proprioceptive guidance. The relative weight of vision and proprioception will depend on their relative precision (variabilities $\sigma_{V}\;\text{and}\;\sigma_{p}$) and on the probability computed by the brain that the virtual hand is one’s own (P_comPrior_). Accordingly, if more weight is given to the visual signal (i.e., the virtual hand) than to the proprioceptive, larger reaching errors are expected in the presence of visuo-proprioceptive incongruence and/or more subjective ownership is reported toward the virtual hand (higher values of P_com_). On the opposite, pure proprioceptive guidance is considered as “zero error”, given the instructions of the task to try to reach the target with the real hand. P_com_ values can be inferred from reaching errors by computing the relative weight of vision and proprioception. P_com_ will decrease with increasing incongruence so that the relative weight attributed by subjects to the virtual hand will be high at small values of disparity and gradually decrease with incongruence. Furthermore, Equation 5 implies that if the sensory precision of vision and/or proprioception is reduced (e.g., by aging), P_com_ values will need larger disparities to decrease significantly.

To infer P_com_ σ_v_ and σ_P_ for each subject, we fitted the Bayesian causal inference model on our multisensory reaching task (VPD) using the BADS MATLAB optimization tool (https://github.com/lacerbi/bads;^95^). Following the approach used in previous studies,^30^^,^^33^ we simulated 50000 trials for each spatial disparity. A cleaning of the data for each participant to remove systematic biases in reaching possibly due to tracking or VR calibration was done by subtracting the Observed reaching error, defined as the distance between the position of the target and the position of the real hand, the mean reaching bias at 0° spatial disparity:

$${Normalized\;Error}_{i}={Observed\;Error}_{i}-\sum_{i,\alpha =0}\sum {Observed\;Error}_{i,\alpha }$$

No other pre-processing step was performed on the data.

This same procedure has already been used and validated in previous studies.^30^^,^^32^ However, we decided to further validate the model by comparing the individual parameters σ_V_ and σ_P_ extracted from the multisensory task through the Bayesian Causal Inference model with their unisensory correspondents measured through independent tasks assessing proprioception in proprioceptive static (proprioceptive judgment task; PJ) dynamic (open loop task; OL) and visuo-proprioceptive (Midline Judgment task; MJ) conditions. Previous studies have shown that the most relevant task for model validation is the Proprioceptive Judgment (PJ) which showed the best correspondence between fitted and measured parameters. However, we decided to administrate to the participants all three tasks designed to match the VPD setup, to capture the relevant unisensory components as accurately as possible in our sample. If the task relies on the proposed CI process, we expect to observe a positive correlation between the fitted parameters and at least one of the measured unisensory parameters. Therefore, we assessed whether the parameters extracted from the unisensory tasks positively correlated with the ones extracted from the multisensory task by performing significance tests on Pearson correlation scores.

As expected, and in line with,^30^ the correlation of the σ_P_ fitted by the model with the proprioceptive precision extracted from the PJ (σ_PJ_) was significant [r(44) = 0.554, *p* < 0.001]. No correlations were found between the fitted σ_P_ and the proprioceptive precision extracted from the OL (σ_OL_; [r(44) = 0.18, *p* = 0.23]) and MJ task (σ_MJ,_ [r(44) = 0.24, *p* = 0.21]; the detailed extraction of unisensory parameters is described below (see details on unisensory parameters extraction in the section below).

All the tasks have been described elsewhere.^30^ Participants were asked to sit on a chair keeping their head and trunk aligned while wearing a head-mounted display (HMD). A custom-made software programmed in Unity was used for the tasks.

The PJ task assesses the proprioceptive accuracy of the participants in static condition. During the task, the experimenter passively moved participants’ real hands to one out of 5 possible target positions selected randomly and arranged at 0°, ±10°, ±20°, ±40° with respect to participants’ sternum on an arc with radius equal to each participant maximum reaching distance and centered to the body midline. Participants’ real hand was hidden from their view, and at the first trial of the series, a virtual hand was displaced at +30° (right) or −30° (left). The task was a two-alternative forced-choice where the participants had to report whether the virtual hand they were observing was at the right or left of their real hidden hand. After the first trial, the position of the virtual hand was moved halving the angle and mirroring it in the opposite direction with respect to the participants’ previous answer. The sign of the initial angle was randomized trial by trial. 5 steps converging algorithm was used to determine where the participants perceived their hand to be. The final proprioceptive estimation was computed as the intermediate hand position between the last displayed position in which the algorithm converged and the next position that would have been displayed by the algorithm according to the participants’ last answer. Each target position was tested 4 times in randomized order, for a total of 140 trials. The σ_PJ_ corresponds to the variability in the perceived hand localizations along the different trials.

The OL task assesses the proprioceptive accuracy of the participants in dynamic condition (see the Table in the “sensorimotor and cognitive tasks’ battery” section of the STAR Methods). The procedure of this task is similar to the VPD task, that is participants were asked to do a reaching movement from a starting position to one out of five visual targets arranged at 0°, ±20°, ±40° with respect to participants' sternum and administrated in random order. The targets appeared 1.5 s after the hand was in the starting position and the participants were instructed to perform the movement as soon the target turned from gray to green. Movement of the hand outside the resting position at any time during the initial resting period automatically restarts the trial. Unlike the VPD task, during the OL participants had no visual feedback (no virtual hand displayed) of their hand during the entire duration of the task. Each target position was displayed 10 times for a total of 50 trials for each participant. The σ_OL_ is computed as the variability in proprioceptive error in reaching the target.

The MJ task aims at evaluating the visuo-proprioceptive alignment precision of the participants. On each trial, a white sphere with a 3 cm diameter moved horizontally across the participants’ field of view at a speed of 10°/s, starting from ±45°, ±40°, ±35°, and ±30° from the body midline, on an arc centered on participants’ sternum, with a radius equal to their maximum reaching distance, as in the VPD. Participants must orally report when they felt that the visual cue was aligned with the midline of their body. The experimenter presses a button to stop the sphere, and then the visual target can be subsequently adjusted on the left or the right to match the proprioceptively perceived midline position. The task was repeated for 9 trials and the starting positions of the visual cue were randomized across trials. The σ_MJ_ is computed as the variability in the localization of the position in the space perceived as aligned with the body midline.

In a previous work on stroke patients,^32^ we found that their increased tendency to embody an incongruent virtual hand during the VPD task was associated with the disembodiment of their real hand. Specifically, patients reported lower ownership of the virtual hand at 0 disparity, when it was spatially congruent the real hand. In the present study, we aimed to investigate whether a similar mechanism applies to older adults. To this end, we performed Spearman correlations between the measured proprioceptive variability, assessed with the PJ task, and both the reaching error and the explicit ownership ratings measured during the VPD task at 0 disparity. Additionally, we conducted inter-group comparisons of explicit ownership ratings and reaching error at 0 disparity between HO and HY participants.

#### Sensorimotor and cognitive tasks’ battery

To evaluate the sensorimotor and cognitive factors underlying the bias in arm length perception observed in older adults we asked the participants in this group to perform a battery of sensorimotor and cognitive tasks.^43^

For tactile abilities, we used the Two Point discrimination Task, well-described in literature^96^ and found to be correlated with mBR.^36^ Further, we used quantitative measures embedded in the VPD task (see above), to ensure rigorous and quantitative assessment of different components of proprioception that may be relevant for the BLT^13^: a static component assessed with the PJ task, considering that BLT not implies any motor response; a dynamic component evaluated with the OL task, in line with the hypothesis of a possible link between motor control and BLT; a visual-spatial alignment tested with the MJ similarly to the nature of the BLT where participants have to refer to a visual marker to indicate the perceived location of the occluded body part. The MJ, OL, and PJ have been suggested to be relevant in body perception processing.^30^ The digit span and TMT are among the most used validated tests performed during aging to assess respectively executive and mnemonic functions^48^^,^^49^^,^^97^ that have been found to be relevant for mBR.^38^^,^^81^^,^^82^ The table describe in detail all the tasks.

**Table:** Table. Brief description of tasks exploited to assess cognitive and sensorimotor functions in older adults

Assessed Function	Task Name	Task Description
Sensorimotor	Two Points Discrimination (tactile acuity)^63^	Participants were stimulated on their upper limbs (arm and hand) with two tactile stimuli of varying distances, and they reported whether they perceived one or two points. Adaptive methods were used for stimuli selection, and sensory thresholds were computed.
	The midline judgment task (MJ; to evaluate visuo-proprioceptive alignment)^30^	Participants wearing VR must report when a visual sphere crossing their field of view on an arc centered on the participants’ sternum is perceived as aligned with the midline of their own body.
	The proprioceptive Judgment task (PJ; to evaluate proprioceptive precision in passive condition)^30^	Participants must report the perceived position of their hidden arm passively moved in different positions localized on an arc centered on their sternum while relying only on proprioception.
	The open Loop task (OL; to evaluate proprioceptive precision in dynamic condition)^30^	Participants wearing VR must reach different targets localized on an arc centered on the participants’ sternum while their real hand is hidden from their view.
Cognitive	Digit Span Forward (short-term memory)^98^	Participants were required to repeat a series of numbers in the same order as read aloud by the examiner.
	Trail Making Test (TMT; cognitive flexibility)^99^	TMT part A requires the patient to connect circles in ascending numerical order. TMT part B requires the patient to connect circles in ascending order, alternating between numbers and letters. TMT B-A is an indicator of cognitive flexibility and shifting ability.

More details related to the PJ, MJ, and OL tasks are provided in the paragraph on *Bayesian Causal Inference framework transposition to the Visual Proprioceptive Disparity (VPD) task*.

Related to the Two Points Discrimination Tasks used to investigate the participants’ tactile acuity, we adapted the method used in^63^. Participants were blindfolded while sitting on a chair with their right arm resting in a prone position. We measured the two-point discrimination threshold (2pdt) on the thenar eminence of the right hand, and on the center of the internal forearm along longitudinal orientations, the order of administration of the two body parts was randomized between participants. Subjects were tactilely stimulated using a caliper, and at each trial, their task was just to report whether they perceived one or two touches on the skin. The starting double posts separation was clearly above the 2pdt according to normative values,^100^ and corresponded to respectively 2 cm for the hand and 4 for the forearm. The separation was then reduced progressively by 50% after each set of three successive correct responses. When subjects made an error, the separation was subsequently increased to the midpoint of the current (erroneous) trial and the immediately preceding (correct) trial. An increase of 150% was achieved only in the case where the starting distance (2 or 4 cm) was perceived as 1 point. This procedure was terminated after 5 inversions of the staircase. The mean between the last two distances perceived correctly was taken as an individual threshold. We then confirmed this 2pdt estimate by delivering five double posts at this randomly intermixed separation with five single posts. If subjects made less than 3 errors, the estimated threshold was accepted for experimental testing. Otherwise, the procedure was repeated.

Concerning cognitive tests, we used the Trial Making Test^101^ which is a well-known measure of cognitive flexibility.^102^^,^^103^ Participants were asked to connect in the correct sequence numbers (part A) and letters (part B) as fast as possible. In case of any error, the experimenter asks the subject to perform a correction, before stepping to the following sequence. The time needed to accomplish the tasks is recorded. The difference in time between completion of the two parts (TMT B-A) is an indicator of cognitive flexibility and shifting ability and has been used for the analysis. The data collected were initially normalized within each population, accounting for specific differences among the various populations. This process allowed for the correction of intrinsic variations due to cultural, demographic, and socioeconomic factors between Italian^104^ and French^105^ populations. Data was then normalized across the entire sample to enable comparative analysis. Finally, we asked participants to perform the digit span forward and backward. The tasks traditionally are comprised in the Wechsler Adult Intelligence Scale - WAIS^106^ which is used as a general test for intelligence and has been developed to assess cognitive ability for adults. The Digit span forward requires the participant to repeat numbers in the same order as read aloud by the examiner and it is considered as a good measure of simple short-term memory. The Digit Span backward represents a qualitatively different type of task. It requires the participants to repeat the numbers in the reverse order of that presented by the examiner, and it is traditionally considered as a task relying more upon working memory skills that should be considered separately from digits forward.^107^ For each participant the tasks were always administered by the same experimenter and the equivalent score span, i.e., the maximum number of digits that the participants were able to recall was registered. For each population, normalized data by age group and type of task were utilized and assigned to a percentile classes (i.e., equivalent scores).^98^ This approach was necessary to ensure comparability across the two distinct populations, as raw scores would not have been normalized to the reference population otherwise. The Digit span backward was initially included in the regression model to assess its potential predictive value. However, upon analysis, it was observed that it exhibited minimal variability, with all subjects having the same equivalent score except for one. This lack of variability can lead to issues such as collinearity and may distort the model’s results. Furthermore, the statistical analysis indicated that it was not a significant predictor (*p* = 0.06328). Given these considerations, Digit span backward was excluded from the final model to enhance its robustness and interpretability, ensuring that only variables with meaningful contributions were retained.

### Quantification and statistical analysis

#### VPD statistical data analyses

To investigate differences in the explicit ownership of the participants we fitted a linear mixed model (estimated using REML and BOBYQA optimizer in R) to predict the explicit ownership (consisting of the explicit score provided to the participants at each trial of the last block) with group and disparities (formula: ownership ∼ group ∗ disparities). The model included ID as a random effect (formula: ∼1 | ID). The package report was used to report the models’ results according to best practice guidelines.^108^

The model’s total explanatory power is moderate (conditional R^2^ = 0.14) and the part related to the fixed effects alone (marginal R^2^) is 0.06.

The same procedure and packages were used to investigate and report differences in the implicit ownership of the participants corresponding to the reaching error computed for each participant and each trial of the VPD task (formula: Reaching Error ∼ group ∗ disparity). The model’s total explanatory power is substantial (conditional R^2^ = 0.49), and the part related to the fixed effects alone (marginal R^2^) is 0.35.

To demonstrate that reaching errors at the VPD can be considered a proxy of subjective ownership, we tested the hypothesis that, at the individual trial level, a higher weight attributed to the virtual hand was associated with higher subjective ownership, even at a fixed disparity. To do so, we computed the “residual” subjective ownership, by subtracting from each rating the average rating at the same disparity. Similarly, residual reaching errors were obtained by subtracting from the reaching error its average value for each disparity. To indicate a larger visual weight with positive residual drift and vice versa, residual drift values were multiplied by the sign of the spatial disparity. Zero disparity values were excluded as they yield no meaningful information in this analysis. Then, we tested whether residual error and ownership values were correlated by means of a linear mixed model.

To better understand the bottom-up and top-down components underlying the ownership mechanism of the participants in the section *Factors underlying the age-related distortions in body perception: a fundamental role of proprioception* we analyzed the unisensory precision extracted from the VPD (bottom-up: σ_v_, σ_P,_ and top-down: P_comPrior_) and the unisensory task (σ_PJ_; see previous paragraph to have the details on the variables extractions). First, to investigate differences in the sensory precision and P_comPrior_, between groups we performed a nonpaired Wilcoxon test. Paired t-tests were performed within each group to detect differences in sensory precision between modalities. Finally, Spearman correlations tests were performed to investigate the relation between the proprioceptive precision σ_PJ_ and: i) the sensory variability extracted from the model σ_PJ_, to demonstrate that the sensory variabilities fitted from the model show correspondence with the proprioceptive variability directly observed during a proprioceptive task; ii) subjective ownership ratings, to assess the model’s validity in explaining alterations in BO through unimodal sensory deficits.

#### Body landmarks localization task

The Body Landmarks Localization task used here has been described elsewhere.^18^^,^^25^ To calculate the width and length of the two body parts (hand and arm), we considered the position (real and perceived) of the five landmarks (see Figure 2A). The hand length was calculated as the mean of the distance between the tip of the external wrist and the distance between the tip of the index and the internal wrist. The arm length was obtained by calculating the mean distance between the marker on the internal wrist and the elbow and the distance between the external wrist and the elbow. The hand width was calculated as the distance between the tip of the index and annular fingers, while the arm width was obtained by calculating the distance between the internal and external parts of the wrist. Then, for each participant, we calculated an index of the bias in the perceived dimension with respect to the actual one (estimated dimension^13^^,^^25^), as the ratio between the perceived and the real size for each body part (arm length, arm width, hand length, hand width):

$$Estimated\;Dimension\;\left(ED\right)=\frac{Perceived\;Dimension}{Real\;Dimension}$$

Values below 1 represent an underestimation of the perceived dimension with respect to the real one, and values above 1 indicate an overestimation. Moreover, we computed the Estimated Error index, i.e., an index representing the length/width estimation bias, independently from the underestimation or overestimation. The Estimated Error provides information related to the accuracy of the size estimation, and it is computed as follows:

EstimatedError(EE)=|ED−1|

In other words, the Estimated Error is a measure of the error between the Estimated Dimension (ED) and the correct dimension (i.e., ED = 1). The lowest possible value is 0 and it represents no error in the perception of the estimated length/width. On the opposite, higher values represent higher errors.

#### BL statistical data analyses

To investigate bias in metric body perception we compared the performances between the young and older groups. First, we checked the normality of the distribution of the indexes using the Shapiro-Wilk test. Then we measured the distance between the Estimated Distance Index of the two groups by running an unpaired Student’s t test for independent samples with the software R Studio (R Core Team, 2017, http://www.R-project.org/). If the distributions were not normal, we performed the analyses with the Wilcoxon signed-rank test. The same procedure has been used for the Estimated Error, and Bonferroni corrections were applied to correct for multiple comparisons, and it was achieved by dividing the probability value by the number of tests conducted (i.e., the number of body parts tested).

To evaluate the sensorimotor and cognitive factors underlying the bias in arm length perception observed in older adults we performed a Linear regression with the arm length Estimated Error as the dependent variable and the sensorimotor and cognitive factors associated with aging as regressors. Before running the analysis, we checked the normality distribution of the dependent variable. The digits span backward was excluded by the analysis because it was not variable enough (all the participants were assigned the highest equivalent score except one who received the immediately preceding (and non-pathological) score). To find the subset of variables in the data resulting in the best performing model we performed a Lasso (Least Absolute Shrinkage and Selection Operator) regression. Leave-One-Out Cross-Validation (LOOCV) was used to determine the optimal lambda value for the Lasso regression model. The cv.glmnet function from the glmnet package was utilized with alpha = 1 and nfolds = 23. The optimal lambda value obtained from the cross-validation was used to fit the Lasso model. The resulting coefficients were examined to identify the variables that survived the regularization process. The cross-validation process identified an optimal lambda value of approximately 0.04. The Lasso regression model retained three predictor variables with non-zero coefficients, the final set of variables included after lasso regression is reported in the results section (Table 2).

### Additional resources

This study was pre-registered at bioRxiv (the preprint server for biology), and the pre-registration details are available at https://doi.org/10.1101/2024.07.23.604821.
