# Supplementary material for: Proprioception impacts body perception in healthy aging – Insights from a psychophysical and computational approach
Source: iScience. 2025 Sep 3;28(10):113481. doi: 10.1016/j.isci.2025.113481 (PMC12495090; doi:10.1016/j.isci.2025.113481)
Supplement: Document S1. Table S1 [file mmc1.pdf]

iScience, Volume *28*

Supplemental information

Proprioception impacts body perception in healthy aging – Insights from a psychophysical and computational approach

Gaia Risso, Marion Bieri, Tommaso Bertoni, Isabella Martinelli, Giulio Mastria, Loredana Catinari, Lara Allet, Andrea Serino, and Michela Bassolino

Table S1. Demographic information and inclusion criteria details for the older adults' sample.

Participant	Age	Gender	Handedness	GDP	MOCA FR	MOCA ITA	Weight Loss	Perceived Fatigue	Isometric Arm Grip Strength	Speed of Walk	Level of Physical Activity	Fried Frailty Index
HO 01	73	M	10	2	NA	4	0	0	28.3	4.06	4	1
HO 02	72	F	10	11	NA	4	1	0	26.3	4.42	3	1
HO 03	72	M	10	0	30	NA	0	0	39.5	3.43	6	0
HO 04	81	M	10	0	27	NA	0	0	31.6	3.36	6	0
HO 05	78	F	10	1	27	NA	0	0	15.1	3.35	4	1
HO 06	77	F	10	4	27	NA	0	0	27.3	3.79	4	0
HO 07	73	F	9	3	27	NA	0	0	18	4.03	4	1
HO 08	66	F	10	4	28	NA	0	0	30.3	3.35	4	0
HO 09	83	M	10	6	27	NA	0	0	32.3	3.93	4	0
HO 10	80	F	10	4	26	NA	0	0	21	4.22	2	2
HO 11	65	F	10	7	28	NA	0	0	30	3.29	3	0
HO 12	65	F	10	1	27	NA	0	0	22.7	3.52	4	0
HO 13	72	F	10	6	28	NA	0	0	19.3	3.98	4	0
HO 14	65	M	10	2	30	NA	0	0	34.6	4.02	5	0
HO 15	74	F	10	0	28	NA	0	0	21.3	3.38	5	0
HO 16	83	F	10	2	29	NA	0	0	23.3	6.15	5	1
HO 17	68	F	10	0	29	NA	0	0	20.7	3.3	3	1
HO 18	75	F	9	3	29	NA	0	0	26.3	3.6	4	0
HO 19	73	F	10	0	27	NA	0	0	26.3	2.57	5	0
HO 20	66	M	10	9	NA	3	0	0	28.7	4.15	6	1
HO 21	88	M	10	2	NA	4	0	0	24.3	5.32	3	1
HO 22	74	F	NA	5	NA	4	0	0	13	5.09	5	2
HO 23	75	M	NA	4	NA	4	0	0	29	4.76	5	1

The Handedness column shows the scores obtained by each participant on the Flanders Handedness Survey, The GDP column represents the scoring obtained by each participant on the Geriatric Depression Scale. The MOCA FR, and MOCA ITA columns indicate respectively normative scoring used to assess the overall cognitive performance of the French (FR) and Italian (ITA) speakers with the MOCA. Finally, the various components of the Fried Frailty Index are reported (see main text for the details).
